# Dietary Supplementation with the Microalga *Galdieria sulphuraria* (Rhodophyta) Reduces Prolonged Exercise-Induced Oxidative Stress in Rat Tissues

**DOI:** 10.1155/2015/732090

**Published:** 2015-03-22

**Authors:** Simona Carfagna, Gaetana Napolitano, Daniela Barone, Gabriele Pinto, Antonino Pollio, Paola Venditti

**Affiliations:** Department of Biology, University of Naples “Federico II”, Via Mezzocannone 8, 80134 Naples, Italy

## Abstract

We studied the effects of ten-day 1% *Galdieria sulphuraria* dietary supplementation on oxidative damage and metabolic changes elicited by acute exercise (6-hour swimming) determining oxygen consumption, lipid hydroperoxides, protein bound carbonyls in rat tissue (liver, heart, and muscle) homogenates and mitochondria, tissue glutathione peroxidase and glutathione reductase activities, glutathione content, and rates of H_2_O_2_ mitochondrial release. Exercise increased oxidative damage in tissues and mitochondria and decreased tissue content of reduced glutathione. Moreover, it increased State 4 and decreased State 3 respiration in tissues and mitochondria. *G. sulphuraria* supplementation reduced the above exercise-induced variations. Conversely, alga supplementation was not able to modify the exercise-induced increase in mitochondrial release rate of hydrogen peroxide and in liver and heart antioxidant enzyme activities. The alga capacity to reduce lipid oxidative damage without reducing mitochondrial H_2_O_2_ release can be due to its high content of C-phycocyanin and glutathione, which are able to scavenge peroxyl radicals and contribute to phospholipid hydroperoxide metabolism, respectively. In conclusion, *G. sulphuraria* ability to reduce exercise-linked oxidative damage and mitochondrial dysfunction makes it potentially useful even in other conditions leading to oxidative stress, including hyperthyroidism, chronic inflammation, and ischemia/reperfusion.

## 1. Introduction

To date the most important products of the health food market contain antioxidant substances, since their intake seems to be negatively correlated with the risk of cancer, strokes, and neurodegenerative affections. Moreover, natural antioxidants have been proposed as substituent for the synthetic antioxidants, butylated hydroxyanisole (BHA) and butylated hydroxytoluene (BHT), which are suspected to be responsible for serious side effects such as liver damage and carcinogenesis [1, 2]. Algae are a source of fat soluble as well as water soluble antioxidants [3], so health and pharmaceutical industry are focusing their attention on the potential use of algal mass farming for the production of natural antioxidant mixture.

*Galdieria* (Cyanidiophyceae, Rhodophyta) is a genus of unicellular algae inhabiting hot springs and other low pH environments [4].* G. sulphuraria*, widely diffused in thermal acidic environments of the world, has shown optimal growth conditions at pH 1.5 and temperatures in the range 35–45°C [5]. Moreover,* G. sulphuraria* is able to grow in the dark, utilizing a wide range of carbon sources [6]. It contains high concentration of phycobiliproteins, among which C-phycocyanin (C-PC) is a strong antioxidant which also has positive effects on inflammation and heart and kidney injuries [7]. The antioxidant and therapeutic potential of C-PC are related to their molecular structure similar to bilirubin, a natural antioxidant that protects lipids from oxidation [8]. The high C-PC content found in* G. sulphuraria* strain grown in heterotrophic conditions has suggested that* G. sulphuraria* could be a promising candidate for the production of this pigment [9]. Moreover, the recent finding that* G. sulphuraria* from heterotrophic cultures shows favorable macro- and micronutrient profiles has suggested its use in food preparations rich in bioavailable proteins and dietary fibers [10].

Interestingly,* G. sulphuraria* thrives in acidic hot springs, where sulphur compounds are largely present. The volcanic emissions contain H_2_S, which is progressively oxidized to sulphur and H_2_SO_4_, causing a sharp decrease of pH values. The large occurrence of sulphur compounds in the environment could enhance the intracellular production of glutathione in* Galdieria* as suggested by preliminary data obtained for* G. phlegraea*, another* Galdieria* species inhabiting the same sites (Carfagna et al., unpublished). The high levels of substances with high antioxidant capacity suggest a possible utilization of* G*.* sulphuraria* coming from heterotrophic cultures as ingredient in preparation of healthy foods to improve their antioxidant capacity [10] and, therefore, their ability to counteract oxidative stress conditions which an aerobic organism can undergo.

To test this hypothesis, in the present paper we evaluated the ability of* G. sulphuraria* supplementation to reduce the oxidative stress induced by acute long-lasting exercise in rat tissues. Moreover, because the oxidative damage has been found to be associated with a reduction in tissue respiratory function [11], we evaluated the capacity of* G. sulphuraria* supplementation to preserve the mitochondrial functionality of exercised rats.

## 2. Materials and Methods

### 2.1. Algal Strain and Medium

The* G. sulphuraria *strain 064 was from the ACUF collection of the Department of Biology of the University of Studies of Napoli “Federico II” (http://www.biologiavegetale.unina.it/acuf.html). Allen medium [12] supplemented with (NH_4_)_2_SO_4_ as nitrogen source was adopted. As carbon substrate, glycerol was selected, at 2% (W/V) concentration, a cheap organic source of carbon, also available as a byproduct from biodiesel transesterification processes [13]. For mass culture, 6 cylindrical photobioreactors were used, 1 L bubble glass columns (0.04 m ID, 0.8 m high), covered with aluminum foils [10]. The working volume was set at 0.9 L. Air was sparged at the photobioreactor bottom by means of a porous ceramic diffuser at volumetric flow rate range of 20 nL/h; 0.2 *μ*m filters were adopted to sterilize air flow inlet and outlet. The photobioreactors were housed in a thermostated chamber at temperature of 36 ± 1°C. The growth of* G. sulphuraria* in the cylindrical photobioreactors was divided into three phases: batch culture, which lasted about two weeks (Allen medium with 2% glycerol was supplemented to the culture one time during this phase to restore the initial nitrogen and glycerol concentration); fed-batch culture, which lasted about one month (to prevent nitrogen and glycerol starvation, a fixed volume (90 mL) of ten-fold concentrated Allen medium with glycerol was periodically added to the culture; the integration did not dilute the broth since the added liquid volume balanced the periodic culture sampling); semicontinuous culture, which lasted about three months (a prefixed volume of microalgae suspension—broth and cells—was weekly replaced with fresh medium; the test simulates closely continuous cultures and the average dilution rate was assessed as the ratio between the replaced suspension volume and the photobioreactor working volume (0.9 L)). During the course of the experiment, the variation of the oxygen ppm in the medium was followed with a Hanna HI 9142 oximeter.

Cell dry weight determination was made with duplicate samples of cultures. During the semicontinuous phase of growth the samples collected every week were centrifuged in an ALC pK121 centrifuge at 4000 g for 10 minutes and washed one time with a 0.5 NaCl solution and two times with distilled water, to remove culture medium constituents. Then, supernatants were discarded, and the algae were frozen at −80°C, lyophilized, ground in a mill, and weighed.

### 2.2. Determination of Glutathione (GSH) Content in Algal Extracts

For glutathione analyses samples of the cells (about 100 mL) from semicontinuous cultures were collected by centrifugation (4000 g for 15 min); the packed cells were resuspended in 2 mL of cold extraction buffer containing 0.1 N HCl and 1 mM EDTA and broken by passing through a French pressure cell (11,000 psi). The homogenate was centrifuged at 15,000 g at 4°C for 15 min, and the clear supernatant was used as crude extract. Thiols were reduced at room temperature for 1 h by mixing 400 *μ*L of the supernatants with 600 *μ*L of 200 mM 2(N-cyclohexylamino)-ethanesulfonic acid (CHES) (pH 9.3) and 100 *μ*L of 3 mM DTT. Aliquots (330 *μ*L) were derivatized in the dark for 15 min by adding 20 *μ*L of 15 mM monobromobimane in acetonitrile. The reaction was stopped by adding 250 *μ*L of 0.25% (v/v) methanesulfonic acid and samples were centrifuged for 15 min (14,000 rpm) [14]. Derivatized thiols were separated and quantified by reverse-phase HPLC using the method described by Newton et al. [15]. Quantification was made against a calibration curve for GSH. Thiol levels were expressed as *μ*mol g^−1^ cell. The concentration of protein in algal cells was determined using the Bio-Rad protein assay based on the Bradford method [16] with bovine serum albumin as the standard.

### 2.3. Determination of C-Phycocyanin (C-PC) Content in Algal Extracts

Cells (50 mL of culture) were harvested by low speed centrifugation (4000 g for 5 min) and washed two times in 50 mmol L^−1^ Na-phosphate buffer (pH 7.0) to remove culture medium constituents. The packed cells were resuspended in 5 mL of cold extraction buffer (50 mmol L^−1^ Na-phosphate buffer, pH 7.2) and broken by passing through a French pressure cell (11,000 psi). The homogenate was centrifuged at 15,000 g at 4°C for 15 min, and the clear supernatant was used as crude extract. Cell debris and proteins in crude cell extracts were precipitated by ammonium sulphate (0–50%) at 4°C under stirring overnight. The precipitate was pelleted by centrifugation for 30 min at 15,000 g at 4°C. The pellet was redissolved in 2.5 mmol L^−1^ Na-phosphate buffer (pH 7.0) and desalted on a Sephadex G25 column (Pharmacia PD10). The C-PC content was measured at 618 and 680 nm and the concentration was determined as described by Kursar and Alberte [17]. For the experiments of food intake samples for semicontinuous cultures of* G. sulphuraria* were periodically collected, centrifuged as previously described, and stored at −80°C. Then cells were lyophilized, and the dried material was pulverized with Ika grinder mill.

### 2.4. Animals

**Figure 1:**
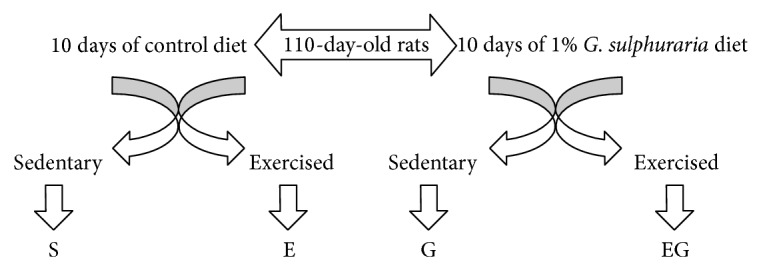
Schematic protocol of rat treatments; 110-day-old animals were randomly assigned to one of two dietary regimens of ten days, receiving either the control diet or a* G. sulphuraria* supplemented diet, consisting of commercial rat chow to which the algae were added to a final concentration of 10 g/Kg. One half of the animals on both the control and supplemented diets were subjected to a 6 h swimming exercise before the sacrifice. The animals swam in a plastic container that was 100 cm high, filled to a depth of 45 cm with water maintained at a temperature between 35 and 36°C. A weight equivalent to the 2% of their body weight was tied to the tail of each rat. Sedentary animals were sacrificed at rest having been kept for 6 h in a small chamber holding about 3 cm of water at 35°C. S: sedentary untreated rats; SG: sedentary* G. sulphuraria* treated rats; E: exercised untreated rats; EG: exercised* G. sulphuraria* treated rats.

The experiments were carried out on 120-day-old male Wistar rats, supplied by Nossan (Correzzana, Italy) at day 100 of age. All rats were subjected to the same conditions (one per cage, constant artificial circadian cycle of 12 h of light and 12 h of darkness, and 50 ± 10% relative humidity) and fed the same diet, a commercial rat chow purchased from Nossan, and water on an* ad libitum* basis. From day 110, animals were randomly assigned to one of two dietary regimens, receiving either the control diet or a* G. sulphuraria* supplemented diet, consisting of commercial rat chow to which the algae were added to a final concentration of 10 g/Kg. The animals were maintained on their respective diets for 10 days. One half of the animals on both the control and supplemented diets were subjected to swimming exercise, so we obtained four rat groups: control sedentary (S), algae fed sedentary (SG), exercised (E), and algae fed exercised (EG) rats (Figure 1). Exercised rats were sacrificed immediately after a 6 h swimming exercise. The animals swam in a plastic container that was 100 cm high, filled to a depth of 45 cm with water maintained at a temperature between 35 and 36°C. A weight equivalent to the 2% of their body weight was tied to the tail of each rat. Sedentary animals were sacrificed at rest having been kept for 6 h in a small chamber holding about 3 cm of water at 35°C.

The treatment of animals in these experiments was in accordance with the guidelines set forth by the University's Animal Care Review Committee.

### 2.5. Tissues Preparation

The animals were sacrificed by decapitation while under anesthesia induced by intraperitoneal injection of chloral hydrate (40 mg/100 g body weight). Exercised rats were sacrificed immediately after the end of the swim session. Liver, heart, and gastrocnemius muscle were excised and placed into ice-cold homogenisation medium (HM) (220 mM mannitol, 70 mM sucrose, 1 mM EDTA, 0.1% fatty acid-free albumin, and 10 mM Tris, pH 7.4). The heart great vessels and valves were trimmed away and the ventricles and atria were cut open and rinsed free of blood. Heart, gastrocnemius muscle (red portion), and liver were freed from connective tissue and the tissues were weighed, finely minced, and washed with HM. Heart and gastrocnemius muscle fragments were incubated for 5 min with HM containing 0.1 mg mL^−1^ nagarse and washed. Finally, all tissues were gently homogenised (20% w:v) in HM using a glass Potter-Elvehjem homogeniser set at a standard velocity (500 rpm) for 1 min. Tissue homogenates were used for analytical procedures.

### 2.6. Preparation of Mitochondria

The homogenates, diluted 1 : 1 with HM, were freed of debris and nuclei by centrifugation at 500 g for 10 min at 4°C. The resulting supernatants were centrifuged at 3000 g for 10 min. The mitochondrial pellets were resuspended in washing buffer (WB) (220 mM mannitol, 70 mM sucrose, 1 mM EGTA, and 20 mM Tris, pH 7.4) and centrifuged at the same sedimentation velocity. Mitochondrial preparations were washed in this manner twice before final suspension in WB. Mitochondrial protein was measured by the biuret method [18].

### 2.7. Oxygen Consumption

Oxygen consumption of homogenates and mitochondria was monitored at 30°C by Hansatech respirometer in 1.0 mL of incubation medium (145 mM KCl, 30 mM Hepes, 5 mM KH_2_PO_4_, 3 mM MgCl_2_, and 0.1 mM EGTA, pH 7.4) with 50 *μ*L of 20% (w/v) homogenate or 0.25 mg of mitochondrial protein per mL and succinate (10 mM), plus 5 *μ*M rotenone, or pyruvate/malate (10/2.5 mM) as substrates, in the absence (State 4) and in the presence (State 3) of 500 *μ*M ADP.

### 2.8. Oxidative Damage

The extent of the lipid peroxidative processes in tissue homogenates and mitochondrial preparations was determined by measuring the level of lipid hydroperoxides (HPS) according to Heath and Tappel [19]. Determination of protein oxidative damage was performed measuring protein-bound (CO) carbonyl levels by the procedure of Reznick and Packer [20] for homogenates and by the modified procedure of Schild et al. [21] for mitochondria.

### 2.9. Antioxidants

Glutathione peroxidase (GPX) activity was assayed at 37°C according to Flohé and Günzler [22] with H_2_O_2_ as substrate. Glutathione reductase (GR) activity was measured at 30°C according to Carlberg and Mannervik [23]. Reduced (GSH) and oxidized (GSSG) glutathione concentrations were measured as described by Griffith [24].

### 2.10. Mitochondrial H_2_O_2_ Release

The rate of mitochondrial H_2_O_2_ release during respiration was measured at 30°C following the increase in fluorescence (excitation at 320 nm, emission at 400 nm) due to oxidation of* p*-hydroxyphenylacetate (PHPA) by H_2_O_2_ in the presence of horseradish peroxidase (HRP) [25] in a computer-controlled Jasko fluorometer equipped with a thermostatically controlled cell-holder. The reaction mixture consisted of 0.1 mg mL^−1^ mitochondrial proteins, 6 U mL^−1^ HRP, and 200 *μ*g mL^−1^ PHPA, in a medium containing 145 mM KCl, 30 mM Hepes, 5 mM KH_2_PO_4_, 3 mM MgCl_2_, and 0.1 mM EGTA, pH 7.4. As respiratory substrates the following were used: 10 mM succinate, plus 5 *μ*M rotenone, or 10 mM pyruvate plus 2.5 mM malate added after 30 seconds of stabilization to start the reaction. Measurements with succinate or pyruvate plus malate in the presence of 500 *μ*M ADP were also performed.

### 2.11. Data Analysis

The data, expressed as mean ± standard error, were analyzed with a two-way analysis of variance method. When a significant* F* ratio was found, the Bonferroni test was used to determine the statistical significance between means. Probability values (*P*) < 0.05 were considered significant.

## 3. Results

The strain 064 of* G. sulphuraria* grown in heterotrophic conditions during the semicontinuous phase of cultivation reached a biomass of 32 g L^−1^ dry weight. In the cells collected during this phase glutathione and C-phycocyanin concentrations of 1.76 ± 0.66 *μ*mol g cell^−1^ and 0.28 ± 0.08 mg g cell^−1^, respectively, were found.

Body weights of rats were 398 ± 5, 396 ± 15, 382 ± 14, and 400 ± 15 g for S, SG, E, and EG groups, respectively, and were not significantly modified by treatments, suggesting that the food intake was the same for all groups and that algal supplementation has no toxic effects.

### 3.1. Oxidative Damage

**Figure 2:**
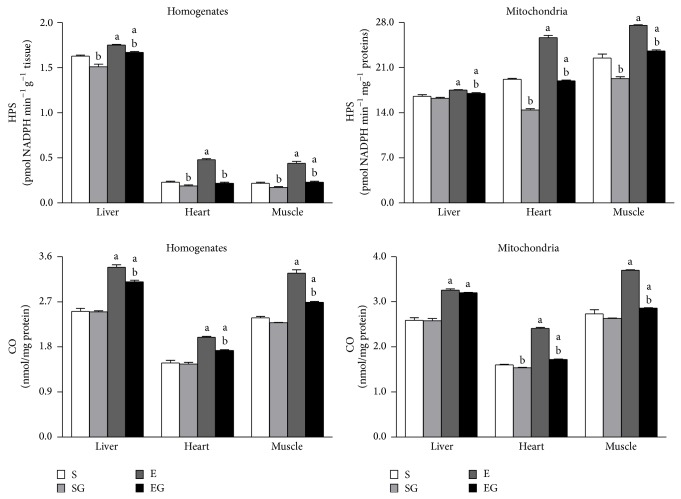
Effect of prolonged exercise and* G. sulphuraria* treatment on the oxidative damage of liver, heart, and skeletal muscle, homogenates, and mitochondria. Values are mean ± S.E.M. For each value eight rats were used. Lipid hydroperoxides (HPS) are expressed as pmol NADPH min^−1^ per g of tissue or mg of mitochondrial protein. Protein-bound carbonyls (CO) are expressed as nmol/mg protein. S: sedentary untreated rats; SG: sedentary* G. sulphuraria* treated rats; E: exercised untreated rats; EG: exercised* G. sulphuraria* treated rats.  ^a^Significant difference for exercised rats versus respective sedentary controls;  ^b^significant difference for* G. sulphuraria* treated animals versus respective untreated controls. The level of significance was chosen as *P* < 0.05.

The levels of lipid hydroperoxides and protein-bound carbonyls are reported in Figure 2. Prolonged aerobic exercise increased the level of lipid hydroperoxide in all tissues of alga unfed rats and in liver and muscle of alga fed rats. Moreover, it increases the levels of lipid hydroperoxide in the mitochondria independently of alga supplementation. Alga supplementation lowered lipid hydroperoxide content in the tissues of both sedentary and exercised rats and in heart and muscle mitochondria of sedentary and in mitochondria of all tissues of exercised rats.

Protein carbonyl content was increased by exercise in tissues and mitochondria of alga treated and untreated rats.* G. sulphuraria* intake reduced protein carbonyl content in heart mitochondria of sedentary rats and in all tissues and in mitochondria from heart and muscle of exercised rats.

### 3.2. Oxygen Consumption

**Figure 3:**
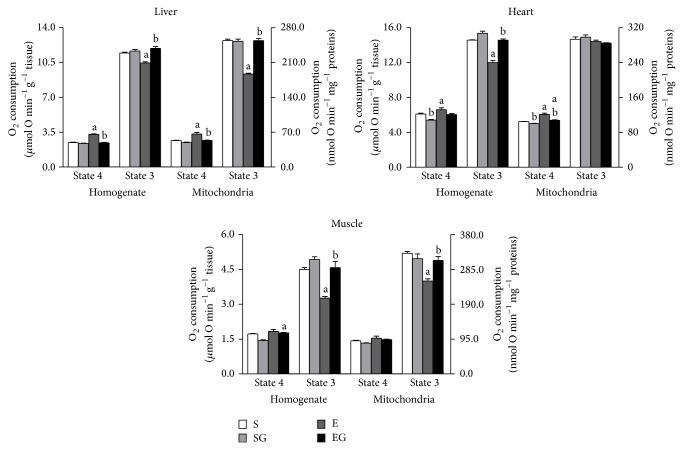
Effect of prolonged exercise and* G. sulphuraria* treatment on the rates of O_2_ consumption of liver, heart, and skeletal muscle, homogenates, and mitochondria, in the presence of Complex II substrate (succinate). Values are mean ± S.E.M. For each value eight rats were used. Oxygen consumption is expressed in *μ*mol O min^−1^ per g of tissue and nmol O min^−1^ per milligram mitochondrial protein. Rates of O_2_ consumption were measured in the absence (State 4) and in the presence (State 3) of ADP. S: sedentary untreated rats; SG: sedentary* G. sulphuraria* treated rats; E: exercised untreated rats; EG: exercised* G. sulphuraria* treated rats.  ^a^Significant difference for exercised rats versus respective sedentary controls;  ^b^significant difference for* G. sulphuraria* treated animals versus respective untreated controls. The level of significance was chosen as *P* < 0.05.

The rates of O_2_ consumption in tissues homogenates and mitochondria in the presence of succinate are reported in Figure 3.

Prolonged exercise increased State 4 oxygen consumption in liver and heart tissues and in mitochondria of alga untreated rats and in heart and muscle tissues and in heart mitochondria from alga treated rats. Exercise lowered the rates of State 3 oxygen consumption in all tissues and in mitochondria from liver and muscle only in alga untreated rats.* G. sulphuraria* supplementation decreased State 4 oxygen consumption in heart and muscle homogenates and in heart mitochondria of sedentary rats and in liver and heart preparations from exercised rats. The State 3 oxygen consumption, which was not changed by* G. sulphuraria* supplementation in sedentary group, was increased by the alga in tissue homogenates and in liver and muscle mitochondria of exercised rats.

**Figure 4:**
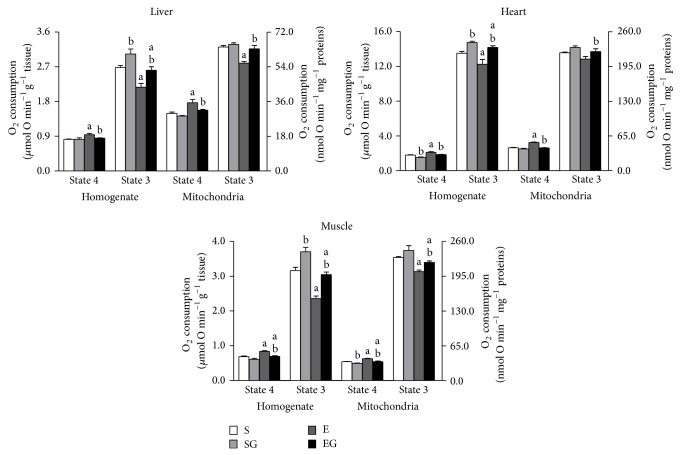
Effect of prolonged exercise and* G. sulphuraria* treatment on the rates of O_2_ consumption of liver, heart, and skeletal muscle, homogenates, and mitochondria, in the presence of Complex I substrates (pyruvate/malate). Values are mean ± S.E.M. For each value eight rats were used. Oxygen consumption is expressed in *μ*mol O min^−1^ per g of tissue and nmol O min^−1^ per milligram mitochondrial protein. Rates of O_2_ consumption were measured in the absence (State 4) and in the presence (State 3) of ADP. S: sedentary untreated rats; SG: sedentary* G. sulphuraria* treated rats; E: exercised untreated rats; EG: exercised* G. sulphuraria* treated rats.  ^a^Significant difference for exercised rats versus respective sedentary controls;  ^b^significant difference for* G. sulphuraria* treated animals versus respective untreated controls. The level of significance was chosen as *P* < 0.05.

In Figure 4 the rates of O_2_ consumption in tissue homogenates and mitochondria in the presence of pyruvate plus malate as substrates are reported.

In the presence of pyruvate/malate prolonged exercise increased State 4 oxygen consumption in all tissues and mitochondria of alga untreated rats and in heart and muscle homogenates and in muscle mitochondria of alga treated rats. Prolonged exercise lowered State 3 oxygen consumption in all tissue homogenates and in liver and muscle mitochondria of alga untreated rats. In alga treated rats exercise lowered State 3 respiration in liver and muscle tissues and in muscle mitochondria.* G. sulphuraria* intake decreased State 4 oxygen consumption in heart homogenate and in muscle mitochondria of sedentary rats and in all preparations of exercised rats. Moreover,* G. sulphuraria* increased State 3 oxygen consumption in all tissues of sedentary rats and in all tissues and mitochondria of exercised rats.

### 3.3. Mitochondrial H_2_O_2_ Release

**Figure 5:**
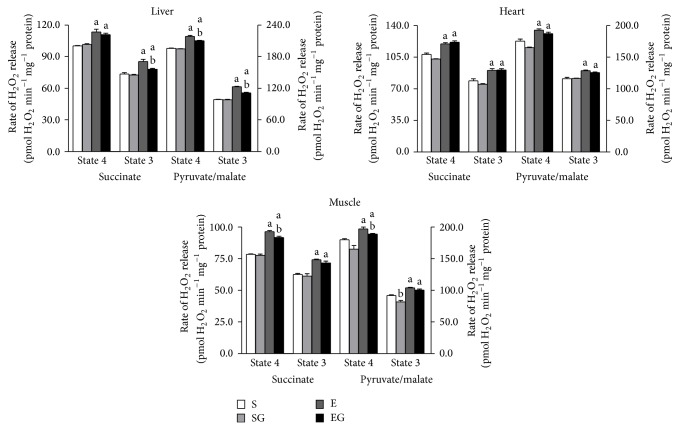
Effect of prolonged exercise and* G. sulphuraria* treatment on the rates of H_2_O_2_ release by rat liver, heart, and skeletal muscle mitochondria in State 4 and State 3 of respiration, in the presence of succinate or pyruvate plus malate, as respiratory substrates. Values are mean ± S.E.M. For each value eight rats were used. Mitochondrial H_2_O_2_ release rate is expressed as pmol min^−1^ per mg of mitochondrial protein. S: sedentary untreated rats; SG: sedentary* G. sulphuraria* treated rats; E: exercised untreated rats; EG: exercised* G. sulphuraria* treated rats.  ^a^Significant difference for exercised rats versus respective sedentary controls;  ^b^significant difference for* G. sulphuraria* treated animals versus respective untreated controls. The level of significance was chosen as *P* < 0.05.

The rates of mitochondrial H_2_O_2_ release are reported in Figure 5. Prolonged exercise increased the rates of both succinate and pyruvate/malate supported H_2_O_2_ release in mitochondria of liver heart and muscle, during both State 4 and State 3 respiration in alga untreated and treated rats.* G. sulphuraria* lowered H_2_O_2_ release in the presence of pyruvate/malate during State 3 respiration in muscle mitochondria of sedentary rats. In exercised group in the presence of succinate as respiratory substrate, the alga consumption lowered H_2_O_2_ release during State 4 in muscle mitochondria and during State 3 in liver mitochondria. In the presence of pyruvate/malate as respiratory substrates, alga lowered H_2_O_2_ release during State 4 in muscle mitochondria and during State 4 and State 3 in liver mitochondria.

### 3.4. Tissue GSH Levels and Antioxidant Enzymes Activities

**Figure 6:**
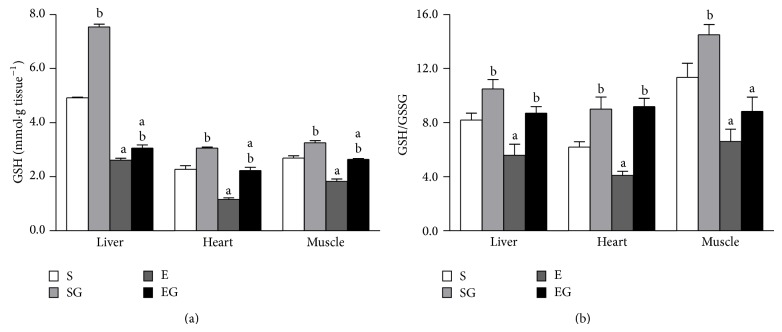
Effect of prolonged exercise and* G. sulphuraria* treatment on rat liver, heart, and skeletal muscle content of reduced glutathione (GSH) (a) and GSH/GGSG ratio (b). Values are mean ± S.E.M. For each value eight rats were used. GSH is expressed as nmol GSH per g of tissue. S: sedentary untreated rats; SG: sedentary* G. sulphuraria* treated rats; E: exercised untreated rats; EG: exercised* G. sulphuraria* treated rats.  ^a^Significant difference for exercised rats versus respective sedentary controls;  ^b^significant difference for* G. sulphuraria* treated animals versus respective untreated controls. The level of significance was chosen as *P* < 0.05.

**Figure 7:**
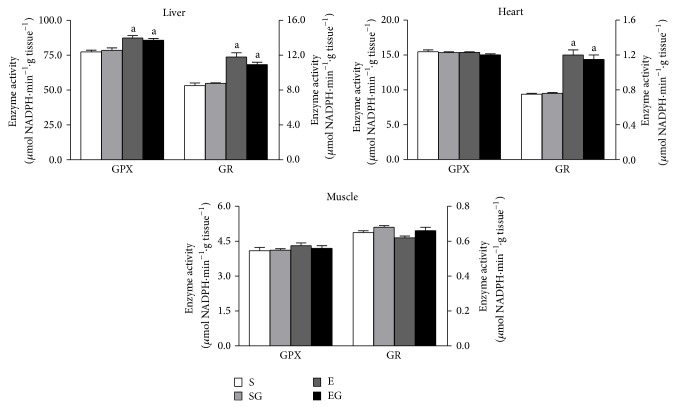
Effect of prolonged exercise and* G. sulphuraria* treatment on rat liver, heart, and skeletal muscle enzyme activities of gluthatione peroxidase (GPX) and reductase (GR) activities. Values are mean ± S.E.M. For each value eight rats were used. GPX and GR activities are expressed as *μ*mol NADPH min^−1^ per g of tissue. S: sedentary untreated rats; SG: sedentary* G. sulphuraria* treated rats; E: exercised untreated rats; EG: exercised* G. sulphuraria* treated rats.  ^a^Significant difference for exercised rats versus respective sedentary controls;  ^b^significant difference for* G. sulphuraria* treated animals versus respective untreated controls. The level of significance was chosen as *P* < 0.05.

The tissue GSH content is reported in Figure 6, upper panel. Prolonged exercise lowered the GSH content in liver, heart, and muscle independently of alga treatment. Alga supplementation increased GSH content in sedentary and in exercised rats. The tissue GSH/GSSG ratio is reported in Figure 6, lower panel. Prolonged exercise reduced the GSH/GSSG ratio in liver, heart, and muscle of alga untreated rats and in muscle of alga treated rats. Alga supplementation increases GSH/GSSG ratio in liver and heart of both sedentary and exercised rats. Moreover, prolonged exercise increased GPX and GR (Figure 7) activities in liver and GR activity in heart, irrespective of algal supplementation.

## 4. Discussion

In physiological conditions antioxidant systems preserve redox homeostasis necessary for normal cell functions, but when free radical and reactive oxygen species (ROS) generation exceeds the cell antioxidant capacity, oxidative stress develops [26] leading to tissue damage and dysfunction.

Since high free radical production during acute exercise was firstly demonstrated by Davies et al. [27], research in the area has greatly grown and it is now clear that the consequent proteins and lipids oxidative damage is responsible for tissues damage, decrease in muscle force production, and fatigue appearance. Conversely, regular exercise induces adaptations which protect against oxidative stress conditions [26] and reduce inflammation [28]. Because the alterations induced by acute exercise are prevented by antioxidant supplementation [29], exercise provides an excellent model to study the dynamic balance between oxidative challenge and antioxidant defense in a biological system. On the other hand, epidemiological and human studies suggest that a diet rich in multiple vitamins is more strongly correlated with a low risk of cancer and other diseases than one rich in an individual vitamin. For such a reason, in the present work, to prevent the exercise-induced oxidative stress, we used dried biomass of* G. sulphuraria *heterotrophic cultures, which has been found to exhibit high antioxidant properties because of its content of antioxidants, including vitamin E and phycobiliproteins [10].

The results reported in the present paper agree with previous reports indicating that acute swimming administration produces increased lipid peroxidation in homogenates from rat muscle, heart, and liver [11, 27, 30, 31] and mitochondria from skeletal muscle [32] and, for the first time, show that exercise also increases lipid peroxidation in mitochondria from liver and cardiac muscle.

Protein oxidation, which was reported to both increase [11, 33] and remain unchanged in homogenates from cardiac and skeletal muscles [34] and liver [33, 34] from running rats, was increased by swimming in all examined tissues and mitochondria. Interestingly, we found that the exercise-induced increase in the lipid oxidative marker in heart and muscle homogenates was about 100%, whereas that found in liver homogenate was lower. This result is likely dependent on the higher antioxidant capacity of the liver in comparison with heart and skeletal muscle [30].

It was previously found that algae supplementation can protect from oxidative stress induced by various pathological conditions. Thus,* Dunaliella salina* reduced the increase in serum levels of a marker of lipid peroxidation, the malondialdehyde (MDA), induced by a diet supplemented with 2% cholesterol [35]. Moreover, the increase in plasma MDA concentration, induced by cadmium administration, was reduced by concomitant* Chlorella vulgaris* supplementation [36].

In this paper we report, for the first time, that* G. sulphuraria* supplementation lowers tissue oxidative damage. However,* G. sulphuraria* supplementation differently affected the levels of lipid and protein oxidative damage in both sedentary and exercised rats. In sedentary rats, the supplementation reduced lipid peroxidation in homogenates and mitochondria but did not affect protein oxidation. In exercised rats, the increase in oxidative damage to lipids was greatly reduced by supplementation, whereas protein oxidative damage was reduced to a lesser extent. These results suggest a specific ability of the alga to preserve lipids from oxidative damage. Such an ability can be attributed to the type of antioxidants contained in* G. sulphuraria* and to the repair processes to which the oxidized lipids are subjected. The C-phycocyanin is able to scavenge various radicals, including peroxyl radicals [37], which are generated during the peroxidative reactions. Vitamin E is the major peroxyl radical scavenger in biological lipid phases. Glutathione, whose oral administration positively influences GSH plasma levels [38], is the cofactor used by the glutathione peroxidase (GPX4) to metabolize the phospholipid hydroperoxides, a process which should lead to their conversion to alcohols and subsequent removal by phospholipase A_2_ [39].

The exercise-induced increase in the oxidative processes was associated with respiration impairment, revealed by an almost general increase in State 4 and decrease in State 3 respiration in all preparations. The increase in State 4 respiration rate represents a compensatory response to the increased leak of protons back into the mitochondrial matrix. The two major pathways of proton leak, the basal proton conductance of the mitochondrial membrane and the inducible proton conductance mediated by specific leak proteins [40], appear to be activated by exercise. Indeed, adenosine monophosphate, whose levels increase during exercise, acts on adenine nucleotide carrier (ANT) to induce H^+^ leak [41]. Moreover, levels of specific uncoupling proteins (UCPs), catalyzing inducible proton conductance, have been found to be increased in cardiac [42] and skeletal muscle [43]. A stronger relationship among exercise, oxidative damage, and proton conductance is supplied by the observation that peroxynitrite, the product of reaction between species generated during exercise, such as superoxide and nitric oxide, increases proton leak enhancing the lipid peroxidation [44].

The observation that reactive oxygen species are able to damage respiratory chain components [45] and that reactive nitrogen species (RNS) inhibit mitochondrial function [46] suggests that the exercise-induced decrease in State 3 respiration is due to a direct action of such species. The ability of the* G. sulphuraria *supplementation to reduce tissue and mitochondrial oxidative damage can explain the attenuation or, in some cases, the prevention of the exercise-induced changes of State 4 and State 3 oxygen consumption in tissue preparations.

It has been proposed that mitochondrial respiratory chain is a major cellular source involved in ROS production during exercise [47]. Respiratory chain produces superoxide anion radical which is converted to hydrogen peroxide (H_2_O_2_) by superoxide dismutase [48]. The hydrogen peroxide escaping antioxidant removal systems is released in the cytosol and converted into hydroxyl radical, which plays a major role in determining the extent of tissue oxidative damage.

We found, according to previous reports [32, 42], that prolonged exercise increases the mitochondrial H_2_O_2_ release rate in cardiac and skeletal muscle. However, we also showed that this also happens in liver. The exercise-induced liver oxidative damage was frequently attributed to ROS production from source different from the mitochondria. Indeed, the exercise and subsequent recovery period appeared to mimic the ischemia/reperfusion phenomenon, in which xanthine dehydrogenase-xanthine oxidase conversion and coupling of xanthine and uric acid formation from hypoxanthine with the univalent oxygen reduction to superoxide are involved. This idea is supported by the observation that liver lipid peroxidation and uric acid content are reduced in exercised rats treated with allopurinol, a xanthine oxidase inhibitor [49]. However, in liver another source of ROS production during reperfusion could be the respiratory chain, which, with resumption of respiration, having high amounts of reducing equivalent and low availability of ADP, should produce ROS at high rate damaging its own components. This, in turn, should block electron flux in some units of respiratory chain, thus increasing ROS production rate. This hypothesis should supply an explanation for the increase in mitochondrial oxidative damage and the higher rate of ROS production found after exercise in isolated liver mitochondria. The fact that* G. sulphuraria *scarcely reduces the exercise-induced increase in mitochondrial ROS production in liver as well as in the other tissues, notwithstanding the reduction in oxidative damage, can be due to the presence in the alga of great amounts of substance able to scavenge peroxyl radicals or metabolize lipid hydroperoxides. Moreover, the reduction of oxidative damage caused by the alga might be the result of its ability to reduce ROS production by a source different from mitochondria or by other antioxidant effects. Indeed, C-phycocyanin inhibits NADPH oxidase expression [50], an enzyme producing O_2_^∙−^ which is found at many sites in skeletal muscle and is activated by muscle contraction [51]. Conversely, it is apparent that the alga does not affect the oxidative damage stimulating the activity of antioxidant enzymes such as glutathione peroxidase and glutathione reductase even though it is not possible to exclude the fact that it can exert an influence on activity of other antioxidant systems.

In conclusion the data here reported indicate that the food supplementation with the alga* G. sulphuraria *protects tissues from the oxidative damage induced by acute exercise. Such a capacity seems to depend on the particular antioxidant content of the alga which contains important sources of glutathione and C-phycocyanin. The latter antioxidant seems to be particularly able to protect lipids from oxidative damage [52]. This suggests the possibility that* G. sulphuraria* supplementation can protect from oxidation plasma lipoproteins reducing cardiovascular risk factors.
